# The clinical behavior and survival of patients with hepatocellular carcinoma and a family history of the disease

**DOI:** 10.1002/cam4.2543

**Published:** 2019-09-18

**Authors:** Jihyun An, Seheon Chang, Ha Il Kim, Gi‐Won Song, Ju Hyun Shim

**Affiliations:** ^1^ Gastroenterology and Hepatology Hanyang University College of Medicine Guri Korea; ^2^ Internal Medicine Myongji Saint Mary's Hospital Seoul Korea; ^3^ Gastroenterology Asan Medical Center University of Ulsan College of Medicine Seoul Korea; ^4^ Asan Liver Center Asan Medical Center University of Ulsan College of Medicine Seoul Korea; ^5^ Surgery Asan Medical Center University of Ulsan College of Medicine Seoul Korea

**Keywords:** clustering, family, liver cancer, prognosis, treatment

## Abstract

**Purpose:**

Familial clustering is a common feature of hepatocellular carcinoma (HCC) as well as a risk factor for the disease. We aimed to assess whether such a family history affected prognostic outcomes in patients with HCC diagnosed at different stages of the disease.

**Materials/Methods:**

This hospital registry‐based cohort study included 5484 patients initially diagnosed with HCC. Individual family histories of cancer were obtained by interview and reported by trained nurses who constructed three‐generation pedigrees. Overall survival data were compared between cases with and without first‐degree relatives affected by HCC, with adjustment for other potential predictors.

**Results:**

Of 5484 patients, 845 (15.4%) had first‐degree relatives with a history of HCC. Family history was associated with longer survival in the entire cohort (adjusted hazard ratio [HR] 0.89, 95% confidence interval [CI] 0.80‐0.98, *P* = .025). A significant trend for reduced risk of death with increasing number of affected family members was also observed (*P* for trend = 0.018). The stage‐stratified analysis showed that the presence of family history was especially associated with a reduced risk of death in the subset of patients with HCC at a (very) early stage (adjusted HR 0.83, 95% CI 0.69‐0.99; *P* = .042). The proportion of cases receiving curative treatment was also higher in early‐stage patients with a family history (72.6% vs 63.3%; *P* < .001).

**Conclusions:**

A first‐degree family history of the disease is a prognostic factor for improved survival in patients with HCC, especially in those whose tumors can be cured by radical treatments.

## Graphical abstract

In this large hospital‐based cohort study, familial clustering of HCC was associated with a reduced risk of overall mortality in patients with the disease. The benefit of a family history was more prominent in early stage HCC cases. A family history was closely associated with initial treatment by curative surgery rather than by non‐surgical options. Our findings suggest that HCC patients with affected relatives seek medical attention more actively and have a higher probability of choosing more effective treatments.

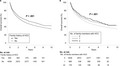

## 1 INTRODUCTION

Evidence has accumulated over many years of a relationship between the risk of developing a specific cancer and a family history of the disease.^1^, ^2^, ^3^, ^4^ In addition, numerous studies have reported positive or negative effects of a family history on the prognostic outcomes of patients with different types of cancer.^5^, ^6^, ^7^, ^8^, ^9^ Most attention in this matter has been given to malignancies of the digestive and reproductive systems, which are the most common cancers in both men and women.^5^, ^6^, ^9^, ^10^, ^11^

Interestingly, hepatocellular carcinoma (HCC), which is the third leading cause of cancer deaths globally despite its lower ranking for incidence, has been observed to cluster within families sharing genes and environments.^12^, ^13^, ^14^ The familial clustering of the disease was found to be unrelated to a viral etiology of hepatitis B in both Asians and Europeans, but understandably increased in subjects with HCC due to vertically transmitted hepatitis B virus (HBV).^13^, ^15^, ^16^, ^17^ Because of this strong familial association of HCC risk, the former American guidelines recommend routine surveillance of hepatitis B carriers of all ages with family histories of HCC, like for high‐risk cirrhotic patients.^18^ An international study of the prognostic role of family history in HCC patients concluded that the familial cancer group had better survival than its sporadic counterpart, and suggested that this was due to the cancers being detected at an earlier stage of tumor growth and liver damage.^19^ However, that (Hong Kong) study gave only unadjusted estimates without detailed information on family membership and generations.

Our aim in this study was to examine the incidence of a family history of cancer at the time of HCC diagnosis, and to investigate the association between familial cancer clustering and survival outcomes over time in a large clinical set of patients first diagnosed with HCC; since the patients were ethnically homogeneous Koreans the data should not be skewed by racial and environmental biases. We also wanted to see whether the presence of affected blood relatives influenced treatment decision‐making in clinical practice.

## 2 PATIENTS AND METHODS

### 2.1 Data sources and collection

Approval of the Institutional Review Board of our center (IRB No. 2016‐0683) was obtained for this large registry‐based retrospective cohort study, and treatment‐naive patients initially diagnosed with HCC by a three‐digit diagnostic code specified by the seventh revision of the International Classification of Diseases (ICD‐7) were identified from our prospectively constructed hospital‐based cancer registry. This registry is a part of the National Cancer Registration Program and has been described in previous studies from our center.^20^, ^21^ Health‐related behaviors together with relevant demographic factors and clinical information on the patients were reviewed from their inpatient and outpatient medical records using the anonymized clinical database system of our institution (Asan BiomedicaL research Environment, ABLE).^22^, ^23^ Demographic and socioeconomic data were collected from computerized admission documents completed by trained nurses during patient interviews employing a structured questionnaire. The nursing charts included information on educational level, substance use (tobacco and alcohol), past and present medical histories, and basic anthropometric data. Medical histories of family members were recorded in detail on each patient's chart, together with pedigrees containing information including history and sites of cancers, and causes of death of close blood relatives (first‐ and second‐degree relatives). We also examined laboratory data related to liver function and viral hepatitis, and checked radiological results to determine stage of HCC based on the size and number of tumors, vascular invasion, and extrahepatic metastasis; in addition, HCC treatment modalities and the associated survival outcomes were obtained from the ABLE system and database of the National Population Registry of the Korea National Statistical Office using the unique personal identification numbers of the patients.

### 2.2 Patient details

**Figure 1:**
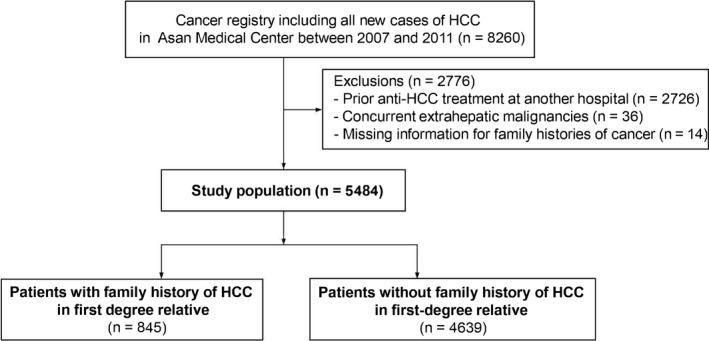
Patient flow diagram

Patients over 20 years of age who were diagnosed as having HCC and underwent treatment for the disease for the first time between 2007 and 2011 were included in this study (n = 8246, Figure 1). Of these, 2762 were initially excluded for the following reasons: 2712 had had previous treatment for HCC prior to visiting our center; 36 had concurrent non‐HCC malignancies; and 14 of whom did not have complete records of family health histories. The diagnosis of HCC was based on either pathological or radiological findings in accord with international guidelines.^18^, ^24^ Cirrhosis of the liver was also defined either histologically or based on radiographic abnormalities (ie, nodular changes of liver morphology, splenomegaly, gastrointestinal varices, or ascites). Stages of HCC at diagnosis were classified by the Barcelona Clinic Liver Cancer (BCLC) system.^24^ The HCC treatment for each patient was principally decided according to their hierarchy of efficacy in lengthening life.

Surgical resection was based on the anatomical segments of the liver whenever possible. Radiofrequency ablation was performed percutaneously under sonographic or computed tomographic guidance. Transarterial chemoembolization (TACE) was usually carried out using a mixture of iodized oil and cisplatin or adriamycin, and absorbable gelatin sponge particles.^25^ Most of transplant cases (97.4%) received grafts from living‐related donors.

### 2.3 Family histories

Family histories of cancer were routinely taken by a trained nurse using a structured questionnaire based on a three‐generation pedigree.^1^ For each relative, the study participant was asked about any serious medical conditions and whether the relative was still alive, or, if the relative had died, the date and cause of death. Positive family histories of cancer were recorded according to type of cancer (HCC or cancer of all types other than HCC) and the generation of the affected relatives (first‐degree or second‐degree). First‐degree relatives included parents, siblings, and offspring; and second‐degree relatives included aunts, uncles, nieces, nephews, and grandparents. A patient with a family history of cancer in both first‐ and second‐degree relatives was regarded as having a first‐degree history. Only histories of cancer in one or more first‐degree relatives that were reliably reported were included as established family histories in the final analysis.^26^

### 2.4 Statistical analysis

The main aim of the statistical analysis was to compare the overall survival of patients with and without a family history of cancer. The survival analysis was censored on 31 December 2016, and deaths occurring up to that time were considered events. In general, overall survival, rather than progression‐free survival, is the most appropriate end‐point of studies of HCC patients, most of whom have underlying liver disease, or some other serious disorder, especially as patients receive different types of curative and non‐curative anti‐cancer treatments.^27^, ^28^, ^29^ Using multivariate Cox proportional hazards models, we estimated the hazard ratio (HR) for death of the familial group compared with the sporadic group as the reference control. The HRs were adjusted for age, gender, level of education, body mass index (BMI), smoking, alcohol consumption, etiology of liver disease, presence of cirrhosis, laboratory results related to liver function, tumor stage at diagnosis, and serum alpha‐fetoprotein (AFP). A backward elimination approach involving candidate variables with *P*‐values < .10 in the univariate analysis was used in the multivariable analysis. Differences in clinical and pathologic parameters between the familial and sporadic groups were analyzed with the *X* ^2^ test or Fisher's exact probability test, as appropriate.

Stratified analyses were also performed by number of affected family members, and cancer stage. A two‐sided *P*‐value < .05 was considered statistically significant.

## 3 RESULTS

### 3.1 Family histories of study subjects

Of 5484 HCC patients included, 1859 (33.9%) had at least one relative with some form of cancer (Table S1); 1823 had family histories in first‐degree relatives and 36 in second‐degree relatives.

When the family history was limited to HCC, 870 (15.9%) had a family history of HCC. 845 (15.4% of the entire cohorts) had family histories in one or more first‐degree relatives and 25 in second‐degree relatives. Nine of the 870 patients had both first‐ and second‐degree family histories of HCC. A total of 1213 patients (22.1%) had family histories of non‐HCC cancers.

### 3.2 Demographic and clinical characteristics according to presence or absence of a family history of HCC

Table 1 presents the baseline characteristics of patients and tumors at the time of HCC diagnosis. The median age of the entire subjects was 56 years (interquartile range [IQR], 49‐63 years), and the majority of the patients were male (80.7%) and had HBV infections (80.6%). Liver cirrhosis was observed in 4431 patients (80.8%). Females and younger patients, non‐diabetics, and never‐drinkers were more common among individuals with first‐degree family histories of HCC than among those without such histories (*P*’s < .05; Table 1). Those with first‐degree family histories of HCC also had a higher proportion of non‐HCC cancer histories (*P* = .002). In addition, subjects with HBV or Child‐Pugh class A were more common and hepatitis C virus (HCV) carriers were less common in the familial group (*P*’s < .05). Size and multiplicity of tumors were not associated with a family history of HCC; BCLC stages were similar in the two groups (*P* = .280), and TACE and surgical resection were the most common primary anti‐HCC treatments in both groups. There was also no difference in the time interval between diagnosis of HCC and initiation of treatment in the two groups, this interval being generally less than one month in new cases (*P* = .306) Curative therapies such as resection, transplantation, and local ablation were initially chosen in 49.1% of the patients with a family history, significantly higher than the 44.0% among those without family histories (*P* < .001), and the converse was true for non‐curative options (50.9% vs. 56.0%; *P* < .001; Table 1).

**Table 1:** Demographic and Hepatic Characteristics by Family History of Hepatocellular Carcinoma (n = 5484)

Variable	Family history (n = 845)	No family history (n = 4639)	*P* value	
Demographic factor				
Male sex	656 (77.6%)	3768 (81.2%)	.015	
Age, years	54 (49‐61)	56 (49‐64)	<.001	
Body mass index, kg/m^2^	24.1 (22.1‐26.1)	24.1 (22.1‐26.1)	.608	
Alcohol consumption			.007	
Never	323 (38.2%)	1515 (32.7%)		
Former	360 (42.6%)	2140 (46.1%)		
Current	162 (19.2%)	984 (21.2%)		
Smoking status			.201	
Never	348 (41.2%)	1762 (38.0%)		
Former	339 (40.1%)	1959 (42.2%)		
Current	158 (18.7%)	914 (19.8%)		
Education, years			.143	
≤9	310 (36.7%)	1857 (40.0%)		
10‐12	298 (35.3%)	1597 (34.4%)		
>12	237 (28.0%)	1185 (25.6%)		
Diabetes	142 (16.8%)	975 (21.1%)	.005	
Hypertension	201 (23.8%)	1250 (27.0%)	.052	
Family history of non‐HCC cancers	219 (25.9%)	977 (21.1%)	.002	
Liver disease‐related factor				
Etiology of liver disease				
Hepatitis B virus infection	762 (90.2%)	3347 (72.1%)	<.001	
Hepatitis C virus infection	28 (3.3%)	513 (11.1%)	<.001	
Liver cirrhosis	690 (81.7%)	3741 (80.6%)	.491	
Ascites	87 (10.3%)	562 (12.1%)	.132	
Platelet count (×10^3^/mm^3^)	143 (98‐190)	137 (94‐189)	.416	
Serum albumin (g/dL)	3.7 (3.3‐4.0)	3.6 (3.1‐4.0)	<.001	
Serum bilirubin (mg/dL)	1.0 (0.8‐1.4)	1.0 (0.8‐1.5)	.078	
International normalized ratio (INR)	1.08 (1.03‐1.17)	1.09 (1.03‐1.19)	.259	
Serum creatinine (mg/dl)	0.8 (0.7‐0.9)	0.8 (0.7‐1.0)	.256	
Child‐Pugh class			<.001	
Class A	710 (84.0%)	3591 (77.4%)		
Class B	108 (12.8%)	860 (18.5%)		
Class C	27 (3.2%)	188 (4.1%)		
MELD score	8 (7‐9)	8 (7‐10)	.003	
Tumor‐related factor				
Number of tumors			.716	
1	518 (61.3%)	2830 (61.0%)		
2	153 (18.1%)	803 (17.3%)		
≥3	174 (20.6%)	1006 (21.7%)		
Maximal tumor size (cm)	3.8 (2.1‐7.7)	4.0 (2.0‐8.0)	.312	
Infiltrative type of tumor	85 (10.1%)	465 (10.0%)	.975	
Vascular invasion	194 (23.0%)	1209 (26.1%)	.057	
Extra‐hepatic metastasis	91 (10.8%)	552 (11.9%)	.348	
Serum AFP (ng/mL)	58.6 (7.9‐976.7)	54.0 (7.9‐993.7)	.063	
Cancer‐related symptoms	907 (19.6%)	156 (18.5%)	.470	
BCLC stage				
Stage 0	90 (10.7%)	440 (9.5%)	.280	
Stage A	344 (40.7%)	1818 (39.2%)		
Stage B	112 (13.2%)	567 (12.2%)		
Stage C	272 (32.2%)	1626 (35.0%)		
Stage D	27 (3.2%)	188 (4.1%)		
Initial anti‐HCC treatment				
Surgical resection	338 (40.0%)	1538 (33.2%)	<.001	.007*
Local ablation therapy	51 (6.0%)	377 (8.1%)	.037	
Liver transplantation	26 (3.1%)	128 (2.8%)	.607	
Transarterial chemoembolization	361 (42.7%)	2118 (45.7%)	.607	
Radiotherapy	3 (0.4%)	20 (0.4%)	>.999	
Systemic chemotherapy	22 (2.6%)	106 (2.2%)	.573	
Conservative management	44 (5.2%)	352 (7.6%)	.014	

Note

Data are presented as number (percentage) or median (interquartile range).

AFP, alpha‐fetoprotein; BCLC, Barcelona Clinic Liver Cancer; HCC, hepatocellular carcinoma; MELD, model for end‐stage liver disease.

* *P* values for curative (ie, surgical resection, local ablation, and liver transplantation) vs non‐curative treatment.

### 3.3 Effect of a family history of HCC on survival in patients with HCC

**Figure 2:**
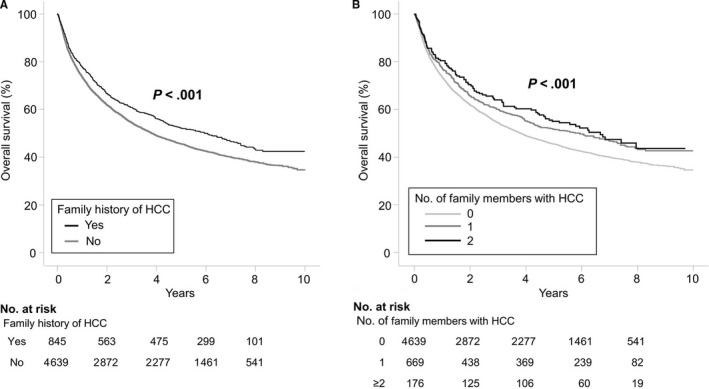
A, Association between presence of family history and overall survival. A first degree family history was significantly associated with longer survival of HCC patients, (B) Overall survival according to number of family members with a history of HCC. There was a significant trend for improved survival with increasing number of affected family members

During a median observation period of 4.0 years (IQR 1.0‐6.6 years), 3228 of the 5484 patients (58.9%) died of any cause. Of those who died, 89.4% (n = 2886) were treated and followed‐up in our tertiary center for at least the last 6 months before death, and this proportion did not depend on the presence or absence of a family history of HCC (91.7% vs 89.0%, *P* = .083). The 3‐, 5‐, and 7‐year estimated overall survival rates were 55.5%, 46.7%, and 41.1%, respectively in the entire population. Kaplan‐Meier log‐rank analysis revealed a significant increase of survival time in the patients with a history of HCC (52.1% vs 45.7% at 5 years, *P* < .001; Figure 2A). In multivariate Cox models after adjustment for co‐predictors (ie, age, gender, family history of non‐HCC cancer, smoking and drinking habitus, level of education, BMI, etiology of liver disease, presence of cirrhosis, Model for end‐stage liver disease [MELD] score, platelet count, serum AFP levels, BCLC stage, and infiltrative type of tumor), a family history of HCC was independently associated with improved overall survival (adjusted hazard ratios [HRs], 0.89; 95% confidence interval [CI], 0.80 to 0.98, *P* = .025; Table 2).

**Table 2:** Effect of family history of HCC on overall survival in the entire population (n = 5484)

Variable	Univariate analysis			Multivariate analysis		
	HR	95% CI	*P*	HR	95% CI	*P*
Family history of HCC	0.83	0.75‐0.92	<.001	0.89	0.80‐0.98	.025
Family history of non‐HCC cancers	0.93	0.85‐1.01	.076	0.98	0.90‐1.07	.695
Male sex	1.26	1.15‐1.38	<.001	1.22	1.11‐1.35	<.001
Age ≥ 60 years	1.16	1.09‐1.25	<.001	1.35	1.25‐1.45	<.001
Current alcohol drinking	1.06	0.98‐1.15	.173	—	—	—
Current smoking habitus	1.14	1.05‐1.24	.002	1.14	1.04‐1.24	.005
Education, years						
≤9	1			1		
10‐12	0.91	0.84‐0.99	.019	0.94	0.87‐1.02	.134
>12	0.74	0.68‐0.81	<.001	0.81	0.74‐0.89	<.001
Body mass index						
<25.0 kg/m^2^	1			1		
25.0‐29.9 kg/m^2^	0.79	0.73‐0.85	<.001	0.84	0.77‐0.90	<.001
≥30 kg/m^2^	1.07	0.91‐1.26	.428	1.10	0.93‐1.29	.274
Diabetes	1.05	0.97‐1.15	.220	—	—	—
Hypertension	0.94	0.87‐1.02	.114	—	—	—
HBV infection	0.88	0.82‐0.95	.001	1.00	0.91‐1.10	.167
HCV infection	1.25	1.12‐1.39	<.001	1.27	1.14‐1.42	<.001
Liver cirrhosis	1.28	1.17‐1.40	<.001	1.23	1.12‐1.36	<.001
MELD score						
≤8	1			1		
9‐10	1.54	1.41‐1.68	<.001	1.45	1.32‐1.58	<.001
11‐14	2.06	1.87‐2.27	<.001	1.91	1.74‐2.11	<.001
≥15	2.45	2.17‐2.76	<.001	1.56	1.33‐1.81	<.001
Platelet count < 100k/mm^3^	0.98	0.91‐1.05	.541	—	—	—
Serum AFP ≥ 100 ng/ml	2.04	1.91‐2.19	<.001	1.63	1.52‐1.75	<.001
BCLC stage						
Stage 0	1			1		
Stage A	1.68	1.41‐2.01	<.001	1.59	1.33‐1.90	<.001
Stage B	3.48	2.88‐4.20	<.001	3.10	2.56‐3.75	<.001
Stage C	7.11	5.99‐8.45	<.001	5.31	4.44‐6.34	<.001
Stage D	7.59	6.06‐9.50	<.001	5.36	4.12‐6.97	<.001
Infiltrative type of tumor	4.27	3.88‐4.71	<.001	2.24	2.02‐2.48	<.001

Abbreviations: AFP, alpha‐fetoprotein; BCLC, Barcelona Clinic Liver Cancer; CI, confidence interval; HBV, hepatitis B virus; HCC, hepatocellular carcinoma; HCV, hepatitis C virus; HR, hazard ratio; MELD, model for end‐stage liver disease.

The relationship between family history and outcomes according to the number of family members with HCC was also investigated. Although the majority of patients with a family history reported only one affected relative, there was a significant trend for an increased reduction in death risk with increasing number of affected family members after adjustment for demographic and tumoral factors (*P* for trend = .018; Figure 2B).

### 3.4 Survival analysis stratified by stage of HCC

**Figure 3:**
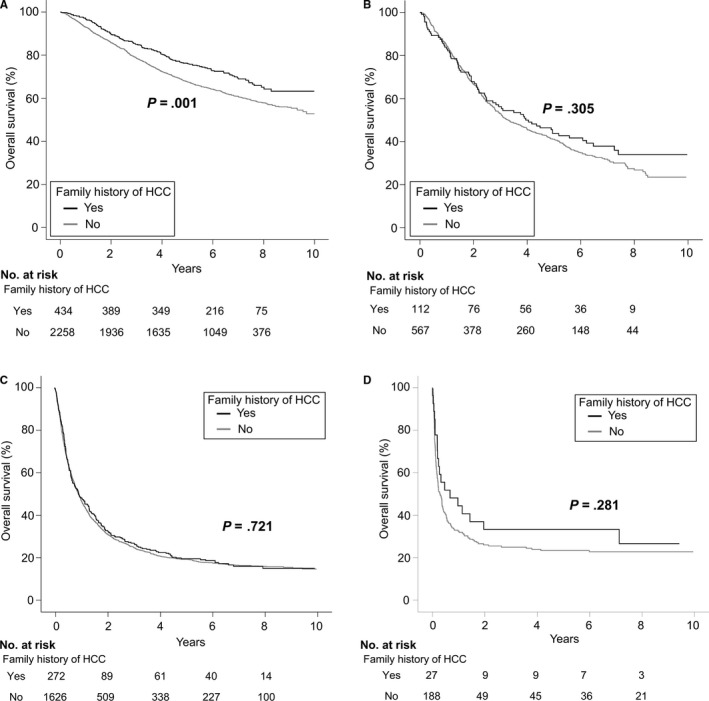
Presence of a family history of HCC and overall survival stratified into BCLC stage ([A] BCLC stage 0 or A, [B] BCLC stage B, [C] BCLC stage C, and [D] BCLC stage D). A family history was associated with better outcomes in patients with BCLC 0‐A stage HCC, but not in those with BCLC stages B‐D

**Figure 4:**
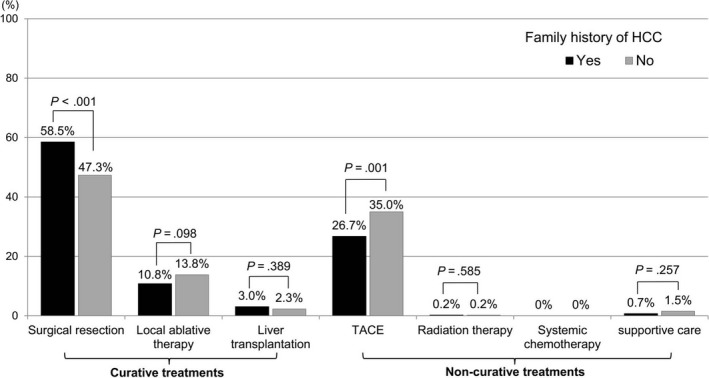
Anti‐HCC treatments according to presence of a family history of HCC in patients with BCLC 0‐A stage HCC. The proportion of patients undergoing curative resection was significantly higher in the group with familial clustering of HCC, whereas treatment with TACE was more prevalent in the sporadic HCC cases

We further analyzed the prognostic effect of a family history of HCC in patients who were at different initial stages of HCC when diagnosed. A family history of HCC was positively correlated with overall survival in patients with BCLC 0 or A stage HCC (adjusted HR 0.83, 95% CI 0.69‐0.99; *P* = .042), as shown in Table S2 and Figure 3. The proportion of cases receiving curative treatment was also higher in early‐stage patients with a family history (72.6% vs 63.3%; *P* < .001). In terms of specific anti‐cancer treatments, surgical resection was more frequently performed in patients with familial histories than in those without histories (58.5% vs 47.3%, *P* < .001; Figure 4). TACE treatment were more common in the latter group (26.7% vs 35.0%, *P* = .001).

When the analysis was restricted to patients with advanced stage HCC, we observed no relationship between survival and family history (HRs [95% CIs] 0.88 [0.68‐1.13] for BCLC B stage; 0.98 [0.85‐1.12] for BCLC C stage; and 0.77 [0.48‐1.24] for BCLC D stage; *P*’s > .05). Among the patients with more advanced HCC, the primary treatment pattern was similar in the two groups (curative vs non‐curative treatments 25.8% vs 24.5%, *P* = .603).

## 4 DISCUSSION

Several studies have demonstrated that a family history of HCC increases the risk of developing HCC, after adjusting for proven risk factors including HBV and HCV infection.^13^, ^17^ Familial aggregation of liver cancer has been frequently reported, although there have been no suggestions of an underlying genetic predisposition for hepatic neoplasms.^12^, ^14^, ^30^ However, the influence of a family history of HCC on subsequent outcomes in patients with the established disease is controversial.^19^, ^31^ In this large, well‐characterized, hospital‐based cohort study, the incidence of a family history in a new HCC series was about 16%, and familial clustering of HCC was associated with a reduced risk of overall mortality in patients with the disease. Curative treatment, especially surgical resection, was also more common in patients with a positive family history.

The role of family history has been investigated as a prognostic factor in several types of malignancy, and diverse correlations have been observed.^6^, ^8^, ^9^, ^32^ The presence of familial cancer in stomach, breast, prostate, and colon cancer patients had protective effects, as in our HCC series, but no such effects were seen for brain and ovarian cancers.^6^, ^8^, ^9^ The univariate findings in a study by a group in Hong Kong pointed to better survival in familial HCC patients (who accounted for approximately 10% of the total), especially in an early‐stage non‐metastatic sub‐cohort, a result that appears to resemble the present findings based on a more intensive and less confounded analysis.^19^ Although another Chinese investigation with 12% familial cases did not find a significant relationship between family history of HCC and survival after resection, the fact that it was restricted to surgical patients limits its generalizability.^31^

There are some possible explanations for the association between familial cancer clusters and prognoses. First, cancer patients with a family history may more often present with early‐stage disease, perhaps because they adhere more rigorously to cancer screening through greater awareness of the implications of the disease, as has been found in studies of prostate, breast, and gastric cancer.^9^, ^33^, ^34^, ^35^ However, a family history did not influence the initial profile of tumor stages among our new HCC cases, and the familial effect persisted after controlling for differences in stage. Second, genetic differences in inherent tumor biology between patients with and without a family history may influence cancer mortality.^36^ A Swedish population‐based study found a higher proportion of indolent subtypes in familial leukemia, whereas familial cases of ovarian cancers had a more aggressive course with poorer survival.^6^ Functional genetic or immunologic polymorphisms may well determine not only susceptibility to specific diseases, but also individual responses to cancer treatment.^37^ Third, health‐related behavioral changes including regular physical activity, stopping smoking and drinking, and a healthy diet and nutrition may contribute to the superior disease course, since these factors have been shown to have anti‐cancer effects in HCC.^38^, ^39^, ^40^

The beneficial effect of a family history in our study appeared greater among patients with early cancers. Since, unlike intermediate or advanced HCC, for which there is a single standard treatment, early‐stage HCC can be treated in a variety of ways, from radical resection or liver transplantation with more curative intent, to less potent but more convenient interventional procedures.^18^, ^24^ This association suggests that familial HCC patients are more likely to seek medical attention, a factor which is seldom controllable; they may therefore have a higher probability of, and even a preference for, undergoing more effective, albeit more invasive, treatment.^35^ Patients with no family history may have less understanding of the therapeutic options and prognoses, and less opportunity to consult someone with experience of HCC, which may influence doctor‐patient decision‐making regarding treatment modality. Our results show that the presence of a family history was closely associated with the receipt of formally recommended definitive surgery, rather than palliative TACE, as the initial therapy in equivalent early cases. In addition to accurate medical knowledge of therapeutic risks and benefits, a variety of factors including fear of complications related to surgery, concern about recurrence, and advice from the patient's family are usually involved in the choice of cancer treatment.^41^, ^42^ Our results suggest that such behavioral benefits associated with family history increase with increasing number of affected relatives. The absence of an association between presence of a family history and survival in patients who had opted for and received surgical resection in our and prior Chinese studies may indicate that a family history is mainly influential during clinical decision‐making.^19^

Several limitations of this study deserve comment. First, because we relied on self‐reported family histories, family history status may have been misclassified. However, self‐reported data have repeatedly been shown to be reliable in prior studies.^43^, ^44^ Because the data on family history were collected at the time of first diagnosis of HCC, prior to the initial treatment, any errors in recall should not have influenced the association with patient outcome.^45^ In addition, we tried to minimize ascertainment bias, and thus in the end only tested the familial effect of first‐degree relatives, and the latter are presumably reported quite reliably.^26^, ^46^ Second, differences in adherence to medical management could introduce a certain bias. However, the interval between diagnosis and treatment among all the new cases, and the follow‐up compliance in our center among the patient who ultimately died, was not affected by a family history of HCC during the period of observation, and indeed patient compliance is likely to be more reliable in a high‐volume hospital like ours.^47^

In conclusion, this investigation revealed that patients with HCC who had a first‐degree family history of the disease survived better than those without such a family history. This familial benefit was stronger among early‐stage patients, whose attitudes would have a greater impact on treatment decisions and subsequent outcomes than among those at a later stage. The molecular and genetic factors underlying familial‐clustered HCC remain to be elucidated.

## AUTHOR CONTRIBUTIONS

J An and S Chang contributed to study concept and design, acquisition, analysis and interpretation of data, statistical analysis, drafting of the manuscript, critical revision of the manuscript for important intellectual content. HI Kim and G‐W Song contributed to acquisition of data and critical revision of the manuscript for important intellectual content. JH Shim contributed to study concept and design, interpretation of data, drafting of the manuscript, critical revision of the manuscript for important intellectual content and study supervision.

## Data Availability

Data used in this research are available to other research teams upon request to the corresponding author.
