# Supplementary material for: The clinical behavior and survival of patients with hepatocellular carcinoma and a family history of the disease
Source: Cancer Med. 2019 Sep 18;8(15):6624–33. doi: 10.1002/cam4.2543 (PMC6825981; doi:10.1002/cam4.2543)
Supplement: Supplementary file 1 [file CAM4-8-6624-s001.docx]

**Supplementary Table 1. Family Histories of Cancer in Patients with Hepatocellular Carcinoma**

| **Family history** | **No. of patients (n=5,484)** |
| --- | --- |
| ***Family history of any cancer*** |  |
| Absent | 3,625 (66.1%) |
| Present | 1,859 (33.9%) |
| Degree of relatives with any cancer |  |
| First degree | 1,823 (98.1%) |
| Second degree | 36 (1.9%) |
| No. of relatives with any cancer |  |
| 1 / ≥2 | 1,321 (71.1%) / 538 (28.9%) |
| No. of first degree relatives with any cancer |  |
| 0 / 1 / ≥2 | 36 (1.9%) / 1,305 (70.2%) / 518 (27.9%) |
| ***Family history of HCC*** |  |
| Absent | 4,614 (84.1%) |
| Present | 870 (15.9%) |
| Degree of relatives with HCC |  |
| First degree | 845 (97.1%) |
| Second degree | 25 (2.9%) |
| No. of relatives with HCC |  |
| 1 / ≥2 | 681 (78.3%) / 189 (11.7%) |
| No. of first degree relatives with HCC |  |
| 0 / 1 / ≥2 | 25 (2.9%) / 669 (76.9%) / 176 (20.2%) |
| ***Family history of cancers other than HCC*** |  |
| Absent | 4,289 (78.2%) |
| Present | 1,213 (22.1%) |
| Degree of relatives with non-HCC cancers |  |
| First degree | 1,196 (98.6%) |
| Second degree | 17 (1.4%) |
| No. of relatives with non-HCC cancers |  |
| 1 / ≥2 | 998 (82.3%) / 215 (17.7%) |
| No. of first degree relatives with non-HCC cancers |  |
| 0 / 1 / ≥2 | 17 (1.4%) / 986 (81.3%) / 210 (17.3%) |

HCC, hepatocellular carcinoma.

**Supplementary table 2. Effect of Family History of HCC on Overall Survival in a Stage-Stratified Survival Analysis**

|  | **BCLC 0 or A** | | **BCLC B** | | **BCLC C** | | **BCLC D** | |
| --- | --- | --- | --- | --- | --- | --- | --- | --- |
|  | **Family member with HCC** | | **Family member with HCC** | | **Family member with HCC** | | **Family member with HCC** | |
|  | **Yes** | **No** | **Yes** | **No** | **Yes** | **No** | **Yes** | **No** |
| No. at risk | 434 | 2,258 | 112 | 567 | 272 | 1,626 | 27 | 188 |
| Number of death | 132 (30.4%)† | 887 (39.3%) | 70 (62.5%) | 389 (68.6%) | 227 (83.5%) | 1,359 (83.6%) | 19 | 145 |
| Unadjusted HR | 0.73  (0.61-0.88)† | 1  (reference) | 0.88  (0.68-1.13) | 1  (reference) | 0.98  (0.85-1.12) | 1  (reference) | 0.77  (0.48-1.24) | 1  (reference) |
| Adjusted HR* | 0.83  (0.69-0.99)† | 1  (reference) | - | - | - | - | - | - |

* Multivariate HRs and 95% CIs are adjusted for age, sex, habitus of alcohol and smoking, hypertension, diabetes, body mass index, family history of non-hepatocellular cancers, etiology of chronic liver disease, presence of liver cirrhosis, MELD score, platelet count, serum alpha-fetoprotein, BCLC stage (only BCLC 0-A subgroup), and infiltrative tumor type.

†*P* value<0.05

HCC, hepatocellular carcinoma; BCLC, Barcelona clinic liver cancer; MELD, model for end-stage liver disease.
